# Towards Understanding the Motivators of Sustainable Consumer Behavior—Validation of the Food Eco-Guilt Scale †

**DOI:** 10.3390/nu16213695

**Published:** 2024-10-30

**Authors:** Brigitta Unger-Plasek, Ágoston Temesi, Zoltán Lakner

**Affiliations:** Institute of Agricultural and Food Economics, Hungarian University of Agriculture and Life Sciences, Villányi Street 29-43, H-1118 Budapest, Hungary; temesi.agoston@uni-mate.hu (Á.T.); lakner.zoltan.karoly@uni-mate.hu (Z.L.)

**Keywords:** scale validation, food eco-guilt, item response theory, Mokken scale analysis, Psych package

## Abstract

Background/Objectives: The growing concern about the environmental impacts of consumption has led to the emergence of so-called “eco-guilt”—a psychological construct reflecting the guilt felt by individuals about the environmental consequences of their choices, which plays a prominent role among the factors influencing pro-environmental behavior. Although eco-guilt has already emerged in other service sectors, such as tourism, and general scales exist to measure it, no such scale exists in the context of food consumption. The aim of this research is to develop and validate a scale to measure eco-guilt related to food consumption. Methods: To create the scale in an objective way, we used the Sustainable Development Goals as a framework. Data were collected from university students; a questionnaire was completed online by 367 respondents. The responses were analyzed from several different perspectives, using multiple methods following the principle of triangulation. For the data analysis, the Psych and Mokken packages of R software (version 4.4.0) were used. Results: The constructed scale was based on 13 items. An overview of the reliability of the scale was provided using various indicators (e.g., Cronbach’s α = 0.86, ω_h_ = 0.63, ω_H asymptotic_ = 0.71, and ω_t_ = 0.89). Based on the analyses, we proposed a reduced form with nine items for the measurement of food-related eco-guilt. Conclusions: The results of this research provide a scale to help understand what motivates consumers to make more sustainable consumption choices. Moreover, the scale is relevant to future research focused on understanding how guilt influences future food choices.

## 1. Introduction

In our everyday lives, our consumption is constantly interwoven with duality; that is, both positive (e.g., pleasure, enjoyment, and happiness) and negative (e.g., guilt) emotions that can arise as a result of our choices. This dichotomy in our daily lives is becoming increasingly more pronounced as consumer awareness grows. Both pleasure and guilt can arise in a variety of situations related to food consumption, which have been investigated in a number of previous studies [1,2,3,4,5]. However, when we talk about guilt in general, we are referring to the emotion that arises from the violation of a personal or social norm, while in the case of eco-guilt—the focus of our research—we are talking about the emotion that arises from the violation of environmental norms. The aim of our research is to better understand and measure this essentially negative emotion.

Nothing demonstrates the ambition and demand of consumers for sustainability better than the growing number of sustainable products on the market. In the food market, their importance is paramount; for example, the market for organic, fairtrade, and other sustainability-related food products is growing worldwide, as evidenced by numerous statistics [6,7,8]. There are many reasons why consumers choose such products. However, despite the value of a product’s sustainability or health benefits, consumers do not make their choices based on these values in many cases [9]. With our consumer habits and choices, we can easily find ourselves in situations where we feel that we do not meet certain personal or social standards. This creates a sense of consumer guilt [10], which is exploited by companies from a practical perspective in their communication, often through advertising [11]. This suggests that it is important to understand the role of guilt not only from a consumer perspective, but also from a practical, corporate perspective. The feeling of guilt and its potential can be significant not only for the company as it also affects our consumer behaviors [12]. Given this dichotomy, several studies have made it an important objective to assess how the emergence of guilt influences consumer behavior.

Guilt can help us to change our consumption habits and thus avoid the recurrence of this fundamentally negative feeling, and it can serve as a driving force for decisions that consumers consider to be better [13]. Several researchers have investigated the impact of guilt on food consumption behaviors from various perspectives; for example, understanding the feelings that arise [14], eating habits and circumstances, attitudes of different consumer groups, [14,15,16], and guilt due to the impact on health [16,17,18,19,20] and the environment [21,22,23,24,25,26] have been the main research directions. Steenhuis’s [14] research on the eating habits of college women and the associated guilt revealed that the type and timing of the food they ate affected the level of guilt induced. Guilt regarding the impact on health is a key area that various researchers have considered on a number of occasions [16,19] For example, research by Daly and colleagues [27] has shown that feelings of guilt play a role in adolescents’ food choices. Others dealt with parents’ feelings about the foods their children are allowed to eat, but at the same time, they considered to be inappropriate [15,16,28]. In relation to consumer health, overconsumption- and underconsumption-related eating disorders are also a focus of research attention [17,18,20,29].

However, we must not forget the positive effects of guilt. For example, Mishra and Mishra [30] showed that feelings of guilt can also lead to a reduction in the consumption of unhealthy foods. The other strand of research is academic work on the effects of guilt; that is, studies focused on how consumers change their buying behaviors to cope with feelings of guilt. In this area, the issue of sustainability has been raised in several ways. These focus areas include meat consumption habits [23,24,25], climate change [22,26], and food waste [21,31]. Previous research has shown how guilt affects consumer behavior in different contexts; however, in many cases, it has been built around guilt in general rather than its specific form, eco-guilt, even when the impact on the environment has been examined. Although there is scientific interest in many aspects of guilt related to food consumption, Yu, et al. [32] have pointed out the need for more research on the relationships between food consumption and guilt. To this end, we believe that our study can make a major contribution by creating a scale to measure eco-guilt, specifically in the context of food consumption.

As an area of guilt, the concept of eco-guilt has also been increasingly explored in research. Eco-guilt is a specific manifestation of guilt where consumers feel that they are not complying with environmental standards or are performing polluting activities [33]. Nielsen and colleagues [34] extended this approach, arguing that eco-guilt is more specific than guilt, since in the former case, it is not just a matter of consumers being aware of these norms but also of accepting them. Their results show that someone with a lower level of environmental awareness does not feel guilty if they behave in an unsustainable way. In some cases, eco-guilt has appeared in general terms or in relation to tourism in previous research [35,36,37].

At the same time, eco-guilt has also been brought to the fore for food products and the catering industry from different perspectives, such as making sustainable consumer choices in specific circumstances [38], reducing waste due to feelings of guilt [39], choosing organic or fair trade products [40,41], and changing meat consumption habits [42]. However, these studies also studied guilt in a more general sense, looking at the effects of consumers feeling that they have acted inappropriately and therefore perceive that they harmed the environment, for example.

The aim of our research is to meet the need for a scale for measuring eco-guilt specifically in the food industry. The food industry and other related industries, such as agribusiness, are affecting the planet like never before [43]. It is, therefore, important from scientific, industrial and business perspectives to understand both what influences consumers and how they are influenced towards more sustainable consumption behavior. To the best of our knowledge, no scale to measure eco-guilt related to food consumption has been developed to date. Therefore, our aim was to construct and validate such a scale. In constructing the items, we considered it important that our research did not focus on the impact or effect of guilt but, rather, focused on the construction of a scale system that allows for measurement of the feeling of guilt itself, and the things that trigger this feeling. In the future, the constructed scale could help to build up a more accurate picture of whether there is a link between environmental impact and guilt related to our food consumption habits, and if so, what this link is.

## 2. Materials and Methods

During the data collection phase of the research, a consumer survey method was used.

The questionnaire was administered to students of the Hungarian University of Agricultural and Life Sciences on two campuses, using convenience sampling, between 12 February 2024 and 16 February 2024. Although convenience sampling has its downsides, such as less generalizable responses, non-representativeness and sample bias, it is often still a useful tool [44], and it is also widely used by researchers in scale validation research [45,46,47,48]. During the data collection period, 367 respondents were reached online. The study was approved by the Interim Ethical Committee of the Hungarian University of Agriculture and Life Sciences, Doctoral School of Economic and Regional Sciences (protocol code 2/2024, 9 February 2024). The research has been pre-registered on AsPredicted.org with the number 161,533.

The grouping of the sample by demographic and income characteristics and their distribution in the sample is shown in Table 1.

In designing the food eco-guilt scale, the best practices from Boateng, et al. [49] were applied. Due to the subjective nature of the topic, an objective framework was needed to create the elements. The Sustainable Development Goals (SDGs) set by the United Nations [50] were used as an objective framework for our research, and statements were made for points that fit the research topic. The aim was to maintain an objective measure, but it is expected that how feelings of guilt subjectively influence everyday habits is taken into account. The SDGs set out actions to live in a more sustainable world. Ecological guilt can influence consumer behavior in many ways, so we have considered the SDGs in which the effect of eco-guilt can manifest itself; namely, no poverty, zero hunger, good health and well-being, clean water and sanitation, reduced inequalities, responsible consumption and production, climate action, life below water, and life on land.

The questions were measured using the most popular form of the Likert scale among social science researchers [51], the 5-point scale, with a score of one indicating that the respondent strongly disagreed with the statement and a score of five indicating that the respondent strongly agreed with the statement. The items of the scale designed to measure eco-guilt are presented in Table 2.

After generating the items, we tested the questions with a small group of four experts to ensure that they were clear and understandable. The main focus of the experts was on whether the statements made were consistent, had no ambiguity, and were grammatically correct, and whether the content was related to the focus of the research. This was followed by the collection of a bigger sample (*n* = 367) and analysis of the obtained responses.

### Data Analysis

The collected data were analyzed using the Psych, PsychTools, and Mokken packages in R software (version 4.4.0). To gain a more comprehensive picture of the reliability of the scale the principle of triangulation was followed [52], which highlights the importance of investigating research questions from several angles. Both the Psych and the Mokken package test the reliability of the scale using different criteria. Psych is a multi-focus package with a strong emphasis on the method of scale analysis [53]. Mokken works with an item-selection algorithm through which Mokken scales are generated to assess the reliability of the scale [54]. Therefore, in the first phase of analysis, the Psych R package [53] was used to examine Cronbach’s alpha (and related) coefficients and to perform an omega principal component analysis. The similarity between statements (items) was examined through hierarchical cluster analysis using the Iclust module of the Psych package.

Using the Mokken scale analysis R package [54], the reliability of the scale was examined from other perspectives using non-parametric tests. Using the Mokken R package, in addition to the basic reliability indicators—namely, Cronbach’s alpha, Gutman lambda 2, and Molenaar Sijtsma (MS) statistics—the scalability coefficients (H, H_i_, H_ij_) were examined, and the monotonicity of the scale was assessed using the automated item selection procedure (AISP). Scalability coefficients help to understand whether the scale elements form a scale or not [55]. The AISP helps to make the questionnaire more robust and shorter by grouping statements [56].

**Figure 1:**
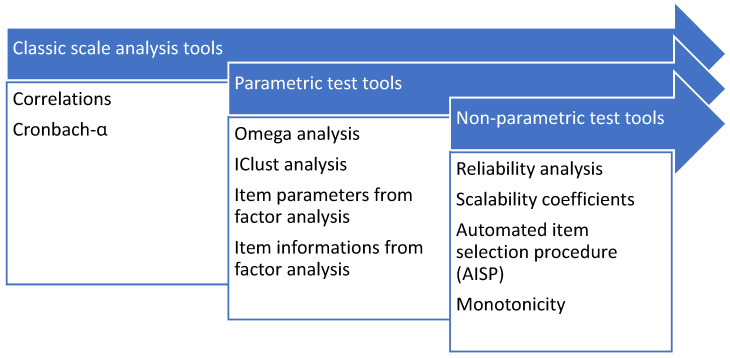
Flowchart of data analysis.

The conducted analyses are summarized in Figure 1.

The combination of different parametric and non-parametric tests allowed for validation of the constructed scale from several points of view, giving a clear picture of its reliability through both examining the relationships between items and the hierarchical structure of the items.

## 3. Results

**Figure 2:**
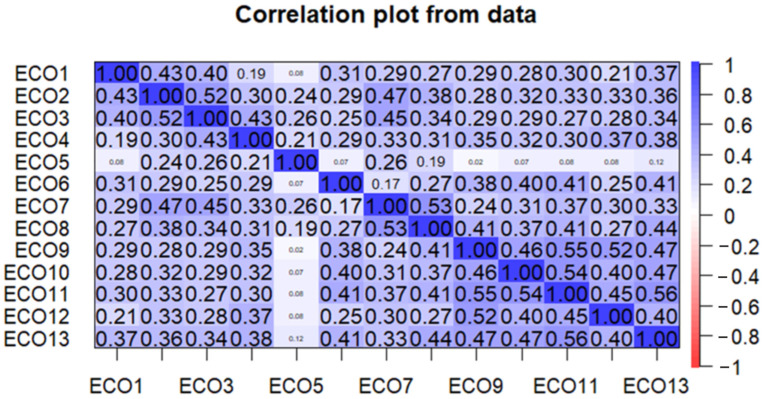
Results of the correlation analysis.

The reliability of the scale was determined through several analyses, which are presented and evaluated below and in the following sections. First, the results of the correlation analysis are presented in Figure 2.

A correlation analysis was carried out to examine the relationships between the items and the convergence of the items. Values greater than 0.5 indicate a strong correlation, while values around 0 indicate that there is no correlation between the items [57]. Figure 2 shows that statement five (ECO5) was weakly correlated with most of the statements (i.e., it had very weak relationships with the other statements). On the other hand, values higher than 0.5, which represent a strong correlation [57], were barely observed in the analysis. Altogether, the results of the correlation analysis indicate that, from a correlation point of view, most of the statements (except ECO5) work well in the scale system.

### 3.1. Test of Cronbach’s Alpha

An important aspect of the scale validation process was to evaluate the internal consistency of the scale system, which was performed using Cronbach’s alpha indicator [58]. The results of the analysis are summarized in Table 3.

Based on the indicators in Table 3, the scale showed good reliability. For both Cronbach’s alpha [59] and Guttmann’s lambda 6 [60], values greater than 0.7 are considered satisfactory, and this criterion was met.

For the Cronbach’s alpha indicators, tests were also carried out to determine whether removing individual items would increase the reliability of the scale. The results are presented in Table 4.

The initial Cronbach’s alpha value was 0.86, against which we compared the results provided in Table 4. In most cases, compared to the previous value, the results did not improve but, instead, worsened. In one case, there was a minimal improvement when the ECO5 statement was removed from the analysis; however, this would not significantly improve the reliability of the scale.

As part of the analysis, the relative frequencies of the responses were obtained, which helps to check how consistently the respondents answered each question. These results are summarized in Table 5.

The relative frequencies shown in Table 5 demonstrated that the responses were spread proportionally between the possible options, inferring the differences in the opinions of respondents. The results in the table show that no statement was answered in the same way by a significant majority of respondents.

### 3.2. Omega Hierarchical Factor Analysis

**Figure 3:**
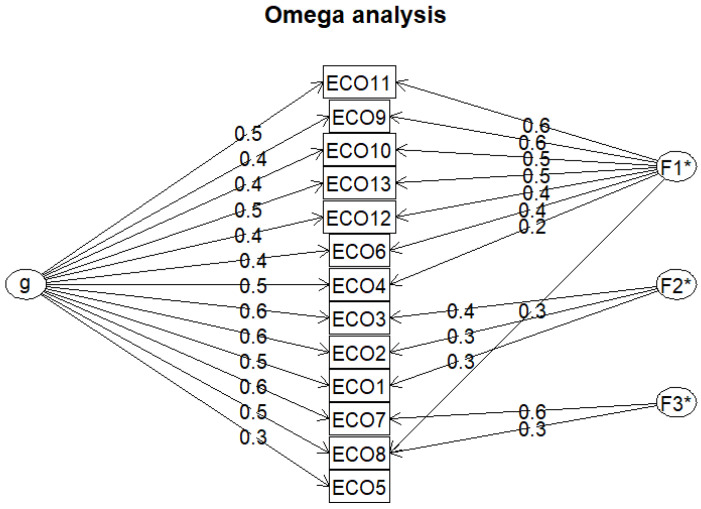
Results of the omega analysis. F1*, F2* and F3* indicates the underlying factors.

The ω_h_ (omega hierarchical) and ω_t_ (omega total) values proposed by McDonald provide an alternative way to test the reliability of a scale and its hierarchical structure [61]. The results of the omega hierarchical factor analysis are presented in Figure 3.

As part of the analysis, we obtained ω_h_, ω_H asymptotic_, and ω_t_ values (ω_h_ = 0.63, ω_H asymptotic_ = 0.71, and ω_t_ = 0.89). Values greater than 0.6 and 0.8 are considered acceptable for ω_h_ values and for the total value, respectively [62]. The results obtained exceeded these values, which also supports the good reliability of the scale. Based on an analysis of Figure 3, statement 5 can be highlighted, which had a lower Schmid–Leiman g-value and was not associated with any of the underlying factors.

### 3.3. IClust Analysis

**Figure 4:**
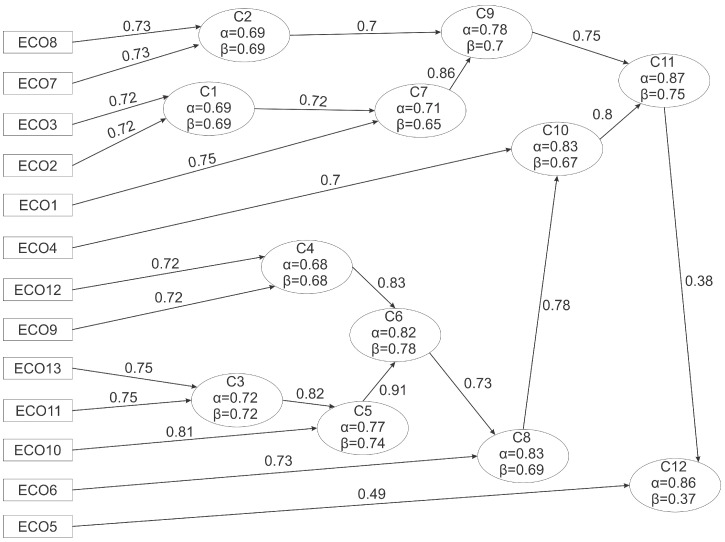
Results of the IClust analysis.

IClust analysis was performed to gain a better understanding of the internal structure of the constructed scale through the clustering of items [63]. The results of the analysis are illustrated in Figure 4.

The dendrogram showing the results of the IClust analysis reveals that a total of 12 clusters were created, in which all the elements are included. The first clusters (1–2) measure attitudes towards products from poor countries (ECO7, ECO8) and the situation of overconsumption (ECO2, ECO3). Together with the food waste claim (ECO1), these clusters formed the higher order cluster 11. The other major branch of the clustering (Clusters 3–6) combines claims related to the environmental impacts of food production and transport, packaging, and water use (ECO9–ECO13). The larger clusters formed from these two main directions eventually led to cluster 12, which compresses all the claims; however, the β value of this cluster was lower, indicating low consistency of the elements within this cluster and overall heterogeneity [63]. While the β values were higher in the previous clusters, inclusion of the ECO5 claim weakened the value considerably, indicating that revision of this claim is required.

### 3.4. Item Parameters from Factor Analysis

**Figure 5:**
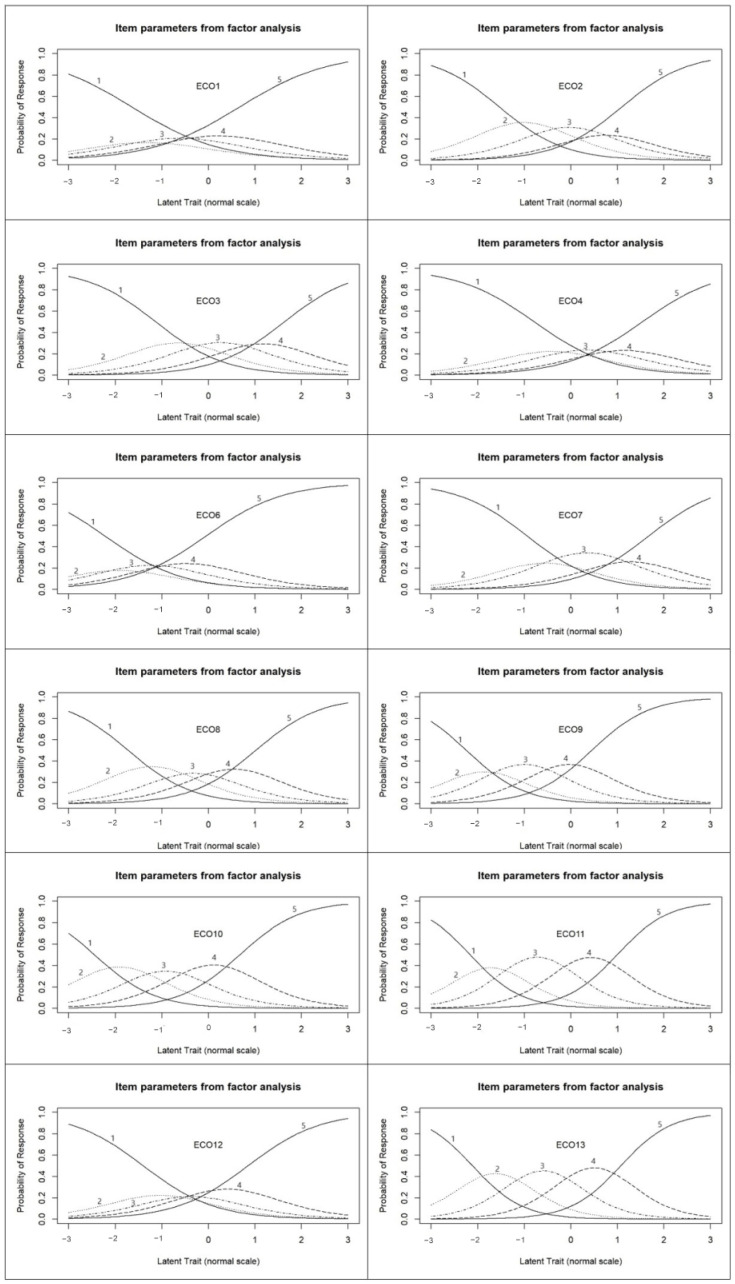
Item parameters from factor analysis. Response option items: 1—totally disagree, 2—disagree, 3—neutral, 4—agree, 5—totally agree.

In order to determine the differences between respondents for each statement, an item parameters from factor analysis was also carried out [64]. This analysis helps to understand how each item contributes to understanding of the latent variable and how it differentiates respondents among the items. The results of the analysis are summarized in Figure 5.

The analysis omitted statement 5 from the outset, as this item was not classifiable in the factor analysis. Looking at the other statements, most were found to discriminate well between respondents, based on how they would respond to each item on a scale of 1–5, as a function of the latent variable (food eco-guilt). However, in several cases, we found that, although the extreme values were clear (those who do not have a food eco-guilt are very likely to give a value of 1 to a given item, while those who do are very likely to give a value of 5), the intermediate values did not sufficiently distinguish respondents in terms of the latent variable.

### 3.5. Item Information from Factor Analysis

**Figure 6:**
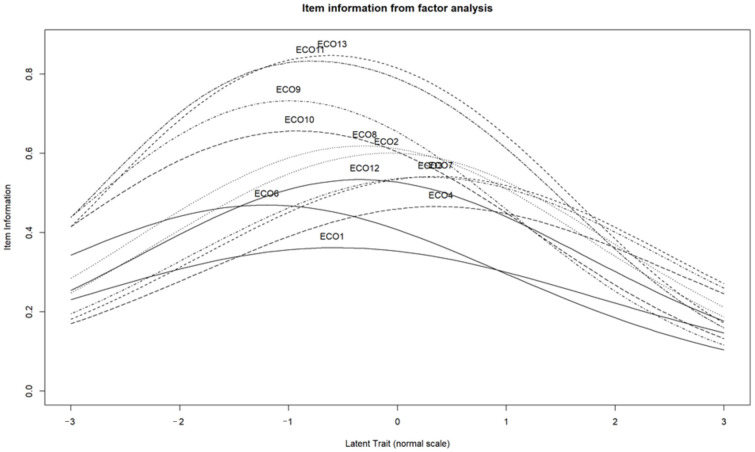
Results of the item information from factor analysis.

For validation purposes, it is also important to determine the information content of each item and the ability to discriminate between respondents at different levels of the latent variable, which can be assessed using item information from factor analysis [61]. The results of this analysis are summarized in Figure 6.

The curves in Figure 6 show the information content of each statement at different levels of the latent variable. An item is considered to be the most informative when the level of endorsement is at least 50% [64]. Based on these scale items, statements 1, 4, and 6 need to be further investigated to increase the effectiveness of the measurement, as these items were found to not adequately measure the differences between respondents at different levels of the latent variable.

### 3.6. Non-Parametric Tests

The Mokken scale analysis method [54] was used to validate the scale with non-parametric tests. In this section, we investigate the scale reliability, scalability coefficients, monotonicity, and the automated item selection procedure (AISP) analysis.

#### 3.6.1. Reliability of the Scale

In the first analysis with the Mokken package, the reliability of the 13-item scale was tested using the Molenaar Sijtsma (MS) [65] indicators, in addition to the previously mentioned Cronbach’s alpha [58] and Guttman lambda 2 [66] indicators.

The values obtained are summarized in Table 6.

The values of the MS indicator reflect the scalability from a non-parametric point of view, as well as the internal consistency of the scale, which indicates good reliability when its value is above 0.7 [67]. Based on the Cronbach’s alpha and lambda 2 values, the scale is reliable. For Cronbach’s alpha, the previously mentioned cut-off value of 0.7 was also taken into account, and for lambda 2 [66], a value greater than 0.8 was considered acceptable.

#### 3.6.2. Scalability Coefficients

An important part of Mokken scale analysis are the values of the scalability coefficients H. The values of the item–pair scalability coefficients (H_ij_), the item scalability coefficients (H_i_), and the test scalability coefficient (H) are summarized in Table 7 and Table 8.

The different values of the scalability coefficients were evaluated based on the work of Van der Ark [68]. Based on the item–pair scalability coefficient values, it can be basically said that, as all values were in the positive range, none of them need to be excluded, and that values greater than 0.3 give positive feedback on scalability, in most cases. However, in several cases, the ECO5 statement showed a weak relationship with other items (with values less than 0.1), indicating that there are weak relationships between these pairs, and the response pattern is inconsistent.

An item scalability coefficient (H_i_) greater than 0.3 may be considered acceptable in order to ensure that statements with too little explanatory power do not impair scalability [68]. In this case, item 5 should be highlighted, for which H_i_ took a value of 0.164.

Based on the test scalability coefficient (H) value, the constructed scale falls in the weak range (between 0.3 and 0.4).

#### 3.6.3. Automated Item Selection Procedure (AISP)

In the AISP, the algorithm creates so-called Mokken scales from the generated scale, during which it can omit items from the original system of scales that cannot be scaled. This procedure yields a scale that satisfies the properties observed using the monotonic homogeneity model and which has sufficient explanatory power [68].

As explained by Koopman, Zijlstra and van der Ark [56], it is worthwhile to consider AISP analysis with increasing lower bound (c) values. Therefore, the results are summarized in Table 9, using different lower bound values.

From the results of the AISP analysis, it is clear at which values the scale diverges and which elements are omitted from the given classification. The AISP analysis and the preceding examination of scalability coefficients are related, and we have already described how the H_i_ values evolved. Given these results, it is not surprising that, even with a small lower bound (c), an element of scalability (ECO5) was dropped. Then, at a value of 0.25, the analysis split the system into two separate scales. The results indicate that the H_i_ coefficients of most items were above 0.3, indicating that the items were well correlated and fit the scale well. However, some items, such as ECO5, had a lower H_i_ coefficient, indicating a poor fit. This demonstrates that this item does not correlate well with the other items and, thus, weakens the reliability of the scale. Overall, while this analysis showed that the constructed scale is consistent and reliable, its level can be further improved through the removal of certain items, namely, ECO1, ECO4, ECO5, and ECO6.

#### 3.6.4. Monotonicity Test

**Figure 7:**
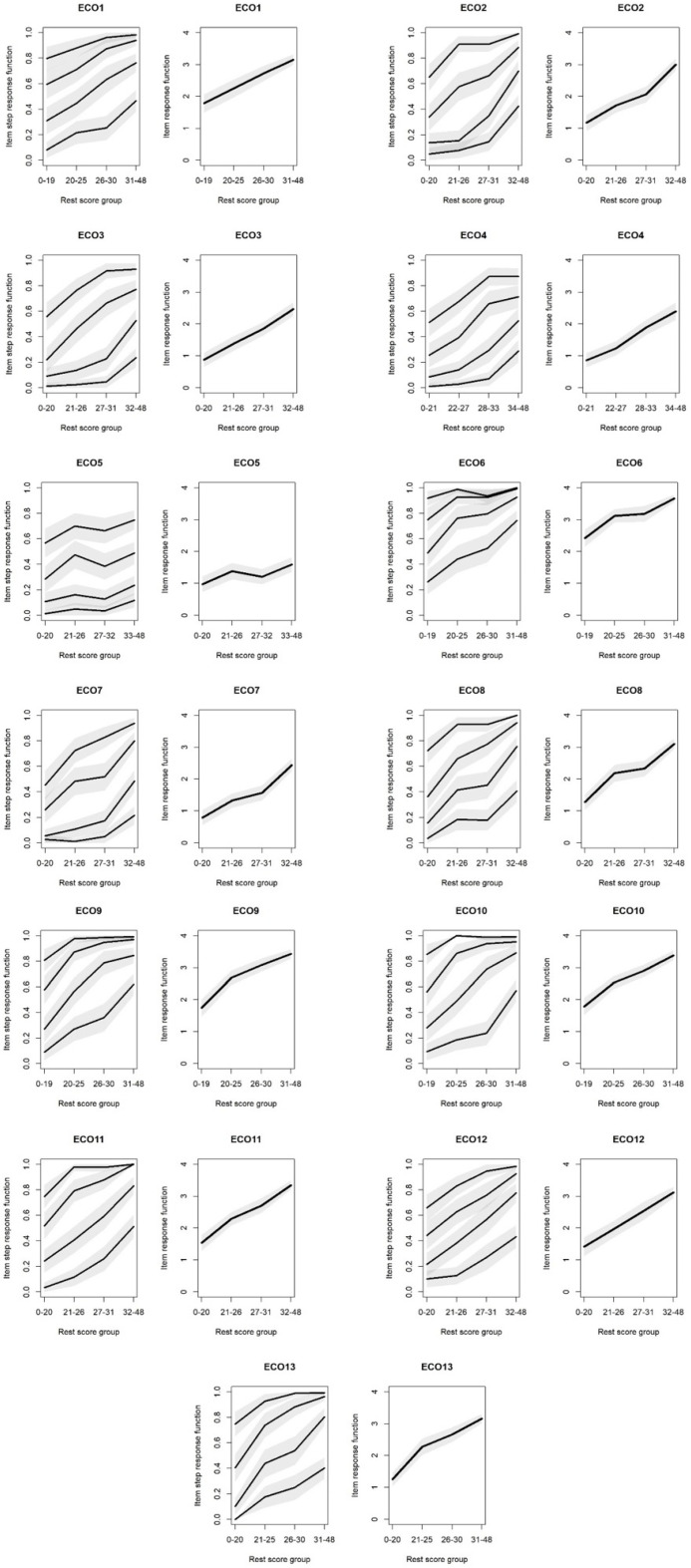
Results of the monotonicity test for each item. The left figure in case of each item indicates the item step response function (from bottom to the top the lines indicate the steps between the answers from totally disagree to disagree, from disagree to neutral, from neutral to agree, and from agree to totally agree), the right one indicated the item response function.

One of the conditions of the Mokken scale is that the monotonicity condition is satisfied. In the next section of our analysis, we examine the monotonicity of each element, the results of which are summarized in Figure 7.

When testing for monotonicity, it is assumed that respondents who have a lower level of environmental guilt about food consumption (low ability level) are more likely to choose the lower response category, while those who have a higher level of environmental guilt are more likely to choose the higher response category.

The results presented in Figure 7 show that, in most cases, the monotonicity condition was satisfied, with the exception of a few items, and the responses increased in line with the values of the latent variable. In three cases, we found that this criterion was violated; namely, for items ECO5, ECO6 and ECO10. For ECO5 and ECO6, larger deviations were observed while, for ECO10, only a minimal decrease was seen in one case, followed by another increase. Based on these results, these three items did not meet the monotonicity criterion of Mokken scalability.

## 4. Discussion and Conclusions

In our research, we used parametric and non-parametric tests to examine the initial 13-item scale, which was framed based on relevant points of the Sustainable Development Goals (SDGs) developed by the United Nations. The results of the analysis revealed that the scale is a reliable and valid instrument for measuring ecological guilt related to food consumption. Overall, both the Cronbach’s alpha and omega values support its suitability. In addition to confirming the one-dimensionality of the model, the omega hierarchical factor analysis also takes into account the underlying factors and examines how the items fit into them. The ECO5 statement here, as in most cases, did not show a good fit, not connecting to any of the underlying factors, which highlights the fact that this statement stands out strongly from the others. The IClust analysis also confirmed that the scale measures one dimension; however, it is important to highlight here that the β value for the last cluster deteriorated considerably after the ECO5 statement was classified. When examining the individual items, in several cases, statement 5 (“I am overly averse to foods that are not common in our country but would help protect the environment”) was not suitable; thus, its omission and modification seem inevitable. The results of the Mokken scale analysis also support this claim: in the AISP analysis, it was already ruled out of the classification by the very first cut-off value. Furthermore, the non-parametric analyses showed that the scale is in the weak range based on the scalability coefficients value, which was strengthened after removing less suitable claims. Such claims were found in other non-parametric tests; three claims did not meet the monotonicity criterion, ECO5 and ECO6 violated this criterion to a greater extent, and ECO10 showed a cask-minimal deviation. In the latter case, since no issue was detected for other measurement criteria, we retained the claim. It is also necessary to fine-tune some other elements of the scale (ECO1, ECO4, and ECO6) in order to further increase the reliability and efficiency of the obtained measurements. Based on our results, the reduced form of the scale, which can be used to measure the food eco-guilt, is summarized in Table 10.

While the use of the reduced scale is recommended, the use of a 1–5-point scale should also be considered for future research. Although this research was not a representative survey and used convenience sampling, the findings that the intermediate items of the 1–5 scale do not sufficiently differentiate respondents in many cases cannot be ignored. The results show that, although the two endpoints of the scale, i.e., 1 (reflecting absolute disagreement with the item) and 5 (reflecting absolute agreement with the item), are capable of discriminating between respondents, the intermediate values yield flatter curves. Based on the item parameters from factor analysis, it is noticeable that the effect of eco-guilt as a latent variable is not significant for statements ECO1, ECO4 and ECO6, as indicated by the flatness of the curves for the intermediate values of the Likert scale (2–4). Based on our results, we would expect that a Likert scale ranging from 1 to 7 would allow for better results and better expression of the respondents’ opinions.

In future research, the proposed scale can greatly contribute to understanding and measuring the motivators of sustainable consumer behavior. Research on this aspect of consumer behavior will have the potential to incorporate a new element into models, which will help to better understand consumer motivators and how guilt is related to sustainable consumer behavior. It will also provide an opportunity to explore the importance of ecological guilt in relation to food consumption using a broad spectrum of methodological approaches.

Today, increasingly more research is focusing on the impact of eco-guilt as an important and significant factor influencing pro-environmental behavior. Several studies have measured eco-guilt from different aspects. Mallett’s [69] research measured eco-guilt with five items dealing with waste recycling, wastefulness, contribution to global warming, use of non-renewable energy and the knowledge that people could do more to minimize their environmental impact. Several studies on eco-guilt have studied these five elements further in other analyses (e.g., [70,71]). Nielsen and colleagues [34] took a similarly general approach in their research, focusing on understanding eco-guilt. Based on deductive and inductive methods, Ágoston and colleagues [33] created a twelve-item scale measuring consumer perceptions of and reactions to climate change and ecological crises (and the eco-guilt in connection with them). In their recent research, Zeier and Wessa [72] developed this further and created a German version of the scale. There are also many studies on the link between eco-guilt and tourism as a priority area [35,36,37,73]; however, research on food consumer behavior is less likely to focus specifically on eco-guilt, and this should be an important aspect of future research.

The results of our research also have limitations from multiple perspectives. First and foremost, the sample may be biased, as we collected data from one country and one segment of consumers using a convenience sampling method. Given the fact that the data were collected from students, a sample that explores the perspectives of other consumer groups may yield different results, which is important to consider in further research. Mallett [69] also points out at the end of his paper that, although this type of sampling is common in psychological research, its generalizability is limited, and the results obtained may vary for nonstudent consumer groups. It is, therefore, important for future research to test the scale on a larger sample (even as far as comparing several countries), so that the data obtained can be better generalized. Nevertheless, as our results show, the Likert scale ranging from 1 to 5 proved to be too narrow, and we could not distinguish between respondents in many respects. To overcome this, future research will need to broaden this range. A third limitation of this research is that, to the best of our knowledge, no other eco-guilt scale has been developed for food, so the proposed scale could not be compared with the results of other validations.

## Figures and Tables

**Table 1:** Demographic and income composition of the sample (*n* = 367).

Category	Sub-Category	*n*	%
Gender	Male	131	35.69
	Female	233	63.49
	Non-binary	3	0.82
Age	18–25	304	82.83
	26–35	35	9.54
	36–45	16	4.36
	46–55	9	2.45
	56 or above	3	0.82
Highest education	Technical school	3	0.82
	High school	298	81.20
	University degree	66	17.98
Place of living	Capital	132	35.97
	Capital agglomeration	64	17.44
	Rural (‘non-agglomeration’) town	114	31.06
	Village/community outside the agglomeration	57	15.53
Perception of income	Very tight	8	2.18
	Tight	24	6.54
	Average	176	47.95
	Good	132	35.97
	Very good	27	7.36

**Table 2:** Items of the food eco-guilt scale and its relation to the SDGs.

Scale Items	Related SDG
I often think about how many people in this world are starving when we throw food out (ECO1)	No poverty, zero hunger
We consume far more calories than we need and others have nothing to eat (ECO2)	Zero hunger, good health and well-being
We eat special food and drink, while others go without it (ECO3)	No poverty, zero hunger, reduced inequalities
We use too much water to prepare our food (ECO4)	Responsible consumption and production
I am overly averse to foods that are not common in our country but would help protect the environment (ECO5)	Responsible consumption and production
We use too much packaging for our products (ECO6)	Responsible consumption and production
Poor countries’ agricultural products do not reach European consumers, so they cannot develop (ECO7)	Reduced inequalities, good health and well-being,
Products from poor countries are often produced with undue exploitation of workers (ECO8)	No poverty, reduced inequalities
We are ruining our environment (e.g., deforesting rainforests) to produce more food (ECO9)	Life on land, climate action
Too many chemicals are used in agricultural production (ECO10)	Life on land, clean water and sanitation
Food production and transport emit too many harmful (greenhouse) gases (ECO11)	Life on land, climate action
If we continue fishing in the sea at this rate, there will not be enough fish left in the sea (ECO12)	Clean water and sanitation, life below water
The transport and storage of food brought from faraway places an unjustified burden on the environment (ECO13)	Climate action

Note: brackets contain the subsequent display of the scale item.

**Table 3:** Results of the Cronbach’s alpha analysis.

Raw Alpha	Std. Alpha	G6 (smc)	Average_r	Mean	sd
0.86	0.86	0.87	0.32	3.3	0.75

**Table 4:** Results of Cronbach’s alpha if item removed.

	Raw Alpha	Std. Alpha
ECO1	0.86	0.86
ECO2	0.85	0.85
ECO3	0.85	0.85
ECO4	0.85	0.85
ECO5	0.87	0.87
ECO6	0.86	0.86
ECO7	0.85	0.85
ECO8	0.85	0.85
ECO9	0.85	0.85
ECO10	0.85	0.85
ECO11	0.85	0.85
ECO12	0.85	0.85
ECO13	0.85	0.85

**Table 5:** The relative frequencies of responses to each statement.

	1	2	3	4	5
ECO1	0.08	0.11	0.23	0.29	0.29
ECO2	0.12	0.23	0.27	0.17	0.21
ECO3	0.19	0.25	0.28	0.18	0.10
ECO4	0.26	0.23	0.24	0.16	0.11
ECO5	0.32	0.26	0.25	0.11	0.06
ECO6	0.04	0.06	0.15	0.24	0.51
ECO7	0.23	0.21	0.31	0.15	0.10
ECO8	0.10	0.20	0.23	0.25	0.22
ECO9	0.06	0.09	0.21	0.27	0.37
ECO10	0.04	0.12	0.22	0.31	0.31
ECO11	0.07	0.12	0.27	0.29	0.25
ECO12	0.13	0.16	0.20	0.26	0.25
ECO13	0.08	0.15	0.26	0.28	0.23

**Table 6:** Reliability indicators determined in the non-parametric test analysis.

MS (Rho)	Cronbach α	Lambda 2
0.8658515	0.8615167	0.8658474

**Table 7:** Item–pair scalability coefficients.

	ECO1	ECO2	ECO3	ECO4	ECO5	ECO6	ECO7	ECO8	ECO9	ECO10	ECO11	ECO12	ECO13
ECO1		0.466 (0.044)	0.420 (0.047)	0.215 (0.057)	0.096 (0.062)	0.334 (0.058)	0.321 (0.055)	0.287 (0.056)	0.308 (0.054)	0.292 (0.053)	0.312 (0.053)	0.226 (0.057)	0.383 (0.053)
ECO2	0.466 (0.044)		0.560 (0.044)	0.327 (0.054)	0.265 (0.057)	0.343 (0.057)	0.507 (0.047)	0.405 (0.050)	0.302 (0.054)	0.339 (0.055)	0.353 (0.056)	0.352 (0.051)	0.391 (0.054)
ECO3	0.420 (0.047)	0.560 (0.044)		0.447 (0.049)	0.284 (0.058)	0.304 (0.056)	0.466 (0.049)	0.369 (0.054)	0.327 (0.058)	0.323 (0.053)	0.289 (0.055)	0.304 (0.054)	0.362 (0.055)
ECO4	0.215 (0.057)	0.327 (0.054)	0.447 (0.049)		0.229 (0.057)	0.358 (0.057)	0.341 (0.054)	0.327 (0.053)	0.407 (0.052)	0.361 (0.050)	0.329 (0.050)	0.397 (0.049)	0.412 (0.051)
ECO5	0.096 (0.062)	0.265 (0.057)	0.284 (0.058)	0.229 (0.057)		0.095 (0.069)	0.281 (0.061)	0.205 (0.057)	0.024 (0.063)	0.082 (0.063)	0.099 (0.063)	0.090 (0.060)	0.139 (0.063)
ECO6	0.334 (0.058)	0.343 (0.057)	0.304 (0.056)	0.358 (0.057)	0.095 (0.069)		0.213 (0.063)	0.303 (0.057)	0.418 (0.059)	0.441 (0.054)	0.453 (0.056)	0.277 (0.059)	0.439 (0.055)
ECO7	0.321 (0.055)	0.507 (0.047)	0.466 (0.049)	0.341 (0.054)	0.281 (0.061)	0.213 (0.063)		0.573 (0.043)	0.277 (0.060)	0.350 (0.059)	0.400 (0.050)	0.318 (0.055)	0.351 (0.055)
ECO8	0.287 (0.056)	0.405 (0.050)	0.369 (0.054)	0.327 (0.053)	0.205 (0.057)	0.303 (0.057)	0.573 (0.043)		0.443 (0.050)	0.396 (0.057)	0.431 (0.051)	0.282 (0.053)	0.459 (0.050)
ECO9	0.308 (0.054)	0.302 (0.054)	0.327 (0.058)	0.407 (0.052)	0.024 (0.063)	0.418 (0.059)	0.277 (0.060)	0.443 (0.050)		0.475 (0.049)	0.591 (0.044)	0.554 (0.045)	0.517 (0.049)
ECO10	0.292 (0.053)	0.339 (0.055)	0.323 (0.053)	0.361 (0.050)	0.082 (0.063)	0.441 (0.054)	0.350 (0.059)	0.396 (0.057)	0.475 (0.049)		0.579 (0.047)	0.428 (0.051)	0.506 (0.050)
ECO11	0.312 (0.053)	0.353 (0.056)	0.289 (0.055)	0.329 (0.050)	0.099 (0.063)	0.453 (0.056)	0.400 (0.050)	0.431 (0.051)	0.591 (0.044)	0.579 (0.047)		0.469 (0.047)	0.574 (0.043)
ECO12	0.226 (0.057)	0.352 (0.051)	0.304 (0.054)	0.397 (0.049)	0.090 (0.060)	0.277 (0.059)	0.318 (0.055)	0.282 (0.053)	0.554 (0.045)	0.428 (0.051)	0.469 (0.047)		0.413 (0.051)
ECO13	0.383 (0.053)	0.391 (0.054)	0.362 (0.055)	0.412 (0.051)	0.139 (0.063)	0.439 (0.055)	0.351 (0.055)	0.459 (0.050)	0.517 (0.049)	0.506 (0.050)	0.574 (0.043)	0.413 (0.051)	

Note: brackets contain the standard errors.

**Table 8:** The item scalability coefficients (H_i_) and the test scalability coefficient (H).

	H_i_	H
ECO1	0.307 (0.032)	0.354 (0.025)
ECO2	0.385 (0.030)	
ECO3	0.375 (0.031)	
ECO4	0.346 (0.031)	
ECO5	0.164 (0.039)	
ECO6	0.335 (0.036)	
ECO7	0.371 (0.031)	
ECO8	0.373 (0.030)	
ECO9	0.391 (0.032)	
ECO10	0.383 (0.032)	
ECO11	0.407 (0.028)	
ECO12	0.343 (0.030)	
ECO13	0.413 (0.028)	

Note: brackets contain the standard errors.

**Table 9:** Results of the automated item selection procedure (AISP).

Item	Lower Bound (c)											
	0.00	0.05	0.10	0.15	0.20	0.25	0.30	0.35	0.40	0.45	0.50	0.55
1	1	1	1	1	1	2	0	0	0	0	0	0
2	1	1	1	1	1	2	2	3	3	0	0	0
3	1	1	1	1	1	2	2	3	3	0	0	0
4	1	1	1	1	1	1	0	0	0	0	0	0
5	1	0	0	0	0	0	0	0	0	0	0	0
6	1	1	1	1	1	1	0	0	0	0	0	0
7	1	1	1	1	1	2	2	2	2	0	0	0
8	1	1	1	1	1	1	2	2	2	0	0	0
9	1	1	1	1	1	1	1	1	1	1	0	0
10	1	1	1	1	1	1	1	1	0	0	0	0
11	1	1	1	1	1	1	1	1	1	1	0	0
12	1	1	1	1	1	1	1	0	0	0	0	0
13	1	1	1	1	1	1	1	1	1	0	0	0

**Table 10:** The food eco-guilt scale.

Scale Items
We consume far more calories than we need and others have nothing to eat (ECO2)
We eat special food and drink, while others go without it (ECO3)
Poor countries’ agricultural products do not reach European consumers, so they cannot develop (ECO7)
Products from poor countries are often produced with undue exploitation of workers (ECO8)
We are ruining our environment (e.g., deforesting rainforests) to produce more food (ECO9)
Too many chemicals are used in agricultural production (ECO10)
Food production and transport emit too many harmful (greenhouse) gases (ECO11)
If we continue fishing in the sea at this rate, there will not be enough fish left in the sea (ECO12)
The transport and storage of food brought from faraway places an unjustified burden on the environment (ECO13)

## Data Availability

The data presented in this study are available on request from the corresponding author. The data are not publicly available due to ethical restrictions.
